# The own-voice benefit for word recognition in early bilinguals

**DOI:** 10.3389/fpsyg.2022.901326

**Published:** 2022-09-02

**Authors:** Sarah Cheung, Molly Babel

**Affiliations:** ^1^Department of Speech-Language Pathology, University of Toronto, Toronto, ON, Canada; ^2^Department of Linguistics, University of British Columbia, Vancouver, BC, Canada

**Keywords:** speech perception, word recognition, bilingualism, speech production, linguistic representation

## Abstract

The current study examines the self-voice benefit in an early bilingual population. Female Cantonese–English bilinguals produced words containing Cantonese contrasts. A subset of these minimal pairs was selected as stimuli for a perception task. Speakers’ productions were grouped according to how acoustically contrastive their pronunciation of each minimal pair was and these groupings were used to design personalized experiments for each participant, featuring their own voice and the voices of others’ similarly-contrastive tokens. The perception task was a two-alternative forced-choice word identification paradigm in which participants heard isolated Cantonese words, which had undergone synthesis to mask the original talker identity. Listeners were more accurate in recognizing minimal pairs produced in their own (disguised) voice than recognizing the realizations of speakers who maintain similar degrees of phonetic contrast for the same minimal pairs. Generally, individuals with larger phonetic contrasts were also more accurate in word identification for self and other voices overall. These results provide evidence for an own-voice benefit for early bilinguals. These results suggest that the phonetic distributions that undergird phonological contrasts are heavily shaped by one’s own phonetic realizations.

## Introduction

Familiar accents and voices receive a range of processing benefits including higher recognition rates, intelligibility boosts, and increased attention in the context of competing speech (e.g., Bradlow and Bent, 2008; Adank et al., 2009; Johnsrude et al., 2013; Holmes et al., 2018). Ones own voice is arguably the most familiar voice, due to our continuous exposure to it. Given that self-recognition, the ability to distinguish between the self and others, is a fundamental human capability, it is therefore unsurprising that self-referential information is processed differently from stimuli associated with others across domains (Keenan et al., 2000; Platek et al., 2004, 2006; Uddin et al., 2005; Keyes et al., 2010; Devue and Brdart, 2011; Zhao et al., 2011; Liu et al., 2019). This extends to voice processing, as researchers have not only observed that people process their own voices differently from others voices (Hughes and Harrison, 2013; Peng et al., 2019; Mitterer et al., 2020), but also that this difference in perception may translate into an advantage in recognizing words in self-produced speech (Eger and Reinisch, 2019).

Spoken language processing is, in a large part, shaped by experience. Infants narrow their perceptual categories based on the language varieties they are exposed to (e.g., Werker and Tees, 1984), and adults prioritize phonetic information in a language-specific manner (e.g., Johnson, 1997; Sumner et al., 2014; Schertz and Clare, 2020). Familiar languages, accents, and voices are afforded benefits in processing, and these benefits surface at different intervals in the pipeline. Concepts like *recognition* (i.e., comprehending the signal) and *encoding* (i.e., updating a representation) are different processes (Clopper et al., 2016; Todd et al., 2019) and consideration needs to be given as to whether any socially skewed or preferential encoding takes place at *perception* or *interpretation* stages (see Zheng and Samuel, 2017). In addition to unpacking the mechanisms by which preferential encoding occurs, the acoustic-auditory substance of *what* is preferentially encoded is not well predicted by theory or supported by consistent empirical results. For example, while there is evidence that familiar speech signals are preferentially encoded (e.g., Clopper et al., 2016), this does not entail that the highest frequency exemplar is the most robustly encoded (Sumner and Samuel, 2005). In some cases, early and consistent experiences shape recognition (e.g., Sumner and Samuel, 2009) and perceptual processing (Evans and Iverson, 2007), whereas in other instances, socially prestigious speech may receive a boost (Sumner and Kataoka, 2013). Familiar accents typically receive benefits, but unfamiliar accents can draw perceptual attention, making them more challenging to ignore than more familiar accents (Senior and Babel, 2018).

The aforementioned examples all relate to accent or dialect differences, but familiarity effects in spoken language are not limited to that level of abstraction. Familiarity effects also extend to individual voices. A large body of research demonstrates that familiarity with a speakers voice eases perception (Nygaard et al., 1994; Newman et al., 2001; Perry et al., 2018). For instance, Nygaard and Pisoni (1998) showed that listeners who successfully learned the voices and names of speakers were better at identifying speech produced by the speakers they were trained on compared to unfamiliar speakers. Evidence of a familiar-talker advantage in perception has been found for young and old listeners (Yonan and Sommers, 2000; Johnsrude et al., 2013), in addition to older listeners with hearing impairments (Souza et al., 2013). Familiar-talker advantages are also found with explicit (Nygaard and Pisoni, 1998) and implicit training (Kreitewolf et al., 2017), as well as in listening conditions with a competing talker in the background (Holmes et al., 2018; Holmes and Johnsrude, 2020). Listeners show improved abilities to selectively attend to or ignore very high familiarity voices (e.g., a spouses voice; Johnsrude et al., 2013), suggesting that a relatively fine-grained prediction is available for familiar voices. Even without awareness of speaker identity, listeners encode acoustically-specific information about words, which can result in more efficient processing if it is similar to existing representations (Creel and Tumlin, 2011).

As noted, an individuals own voice is, arguably, the voice that one has most familiarity with. Importantly, however, self-voice perception of ones own voice sounds different from others because of the different mediums through which sound is physically conducted during perception. When listeners hear their own voices as they speak, sound is transmitted via both air and bone conduction (Shuster and Durrant, 2003; Reinfeldt et al., 2010). In air conduction, vibrations exit the oral cavity, travel through air and enter the ear canal, whereas in bone conduction, vibrations move through the skull bone directly to the cochlea (Stenfelt and Goode, 2005). Comparatively, when listeners hear others speak or hear their own voice in recordings, sound is conducted solely via air conduction. Despite these differences, listeners are very successful at recognizing their own productions in recordings (Xu et al., 2013). Xu et al. (2013) presented listeners with recordings of their own voices and the voices of other, familiar speakers in normal and difficult listening conditions. They found that even in high-pass filter conditions that removed acoustic information from the mean of an individuals third resonant frequency and above, listeners were able to identify their own voices. Researchers theorize that auditory familiarity with ones own voice and the association between auditory self-representation and motor representations may contribute to this self-recognition advantage (Xu et al., 2013).

Beyond an advantage in own-voice recognition, speakers monitor their own productions through auditory feedback. Delayed auditory feedback induces an increase in foreign accent for second language learners (Howell and Dworzynski, 2001) and an increase in regional accent for those who have acquired a different accent (Howell et al., 2006). This suggests that when the timing of auditory feedback is perturbed, individuals are unable to monitor their speech as effectively, resulting in a shift in their speech patterns. Real-time shifts in auditory feedback, where an individual hears resynthesized versions of their own productions that deviate from what they produced, elicits compensation to account for the synthesized acoustic shift (e.g., Houde and Jordan, 2002; Jones and Munhall, 2002; Purcell and Munhall, 2006; Katseff et al., 2012). Crucially, the magnitude of an individuals compensatory response is associated with the shifted items position in the vowel space; shifted items that fall near a phonetic category boundary elicit a larger compensatory response (Niziolek and Guenther, 2013). Compensation for auditory feedback appears to be generally heightened for linguistically relevant dimensions (Xu et al., 2004; Chen et al., 2007; Mitsuya et al., 2011; Niziolek and Guenther, 2013).

While ones own auditory feedback is valuable to the control of motor actions in speech, do ones own productions provide a recognition advantage at the word level? Word recognition can be considered a process that serves to comprehend the speech of *others*, as, under normal contexts, an individual is aware of the linguistic message that is emitted from their own vocal tract. We are interested in how own-voice familiarity shapes the representational and recognition space for linguistic contrasts in word recognition and the acoustic-phonetic distributions that implement phonological contrasts. To test how ones own implementation of a contrast affects word recognition, an introduction of some kind of adverse listening condition is required, as identifying words in a familiar language is a fairly trivial task. Scholars have approached this from two angles with second-language (L2) learners or first language listeners each of which has used relatively distinct methods and landed on different conclusions.

From the L2 perspective is Eger and Reinisch (2019), who demonstrated that German-speaking learners of English were better at recognizing self-produced words in English. This suggests that L2 language learners prioritize their own realizations of phonological contrast. In a related study, Mitterer et al. (2020) show that German-speaking learners of English rate their own, in this case, vocally disguised, sentence productions as more target-like. Mitterer and colleagues offer the interpretation that it is the comprehension advantage afforded by ones own voice that supports higher ratings for self-produced sentences. However, these results for L2 language learners contrast with claims made when processing a first language. For an individuals first language, there is a reported benefit to processing the most statistically average voice over their own self-produced voice when listeners are asked to identify noise-vocoded words, a manipulation that removes fine spectral detail, but spares temporal cues and amplitude modulation (Schuerman et al., 2015, 2019). There is, however, some evidence that L1 listeners word recognition in sentences masked with speech-shaped noise shows a benefit for self-produced sentences compared to sentences produced by others (Schuerman, 2017). Schuerman et al. (2015, 2019) suggest that listeners preferred linguistic representations are informed by the input perceived in ones speech community hence the improved recognition for the statistically average voice in noise-vocoded speech. They reason that own-voice preferences may only arise when listeners are aware that they are hearing their own voice, which is challenging in noise-vocoded speech. The mechanism for the own-voice benefit for L2 English learners posited by Eger and Reinisch (2019) presumes that an individual recognizes their own voice and then further perceptually adapts to their own productions.

In the current study, we test the own-voice benefit for word recognition in early bilinguals, leveraging the high levels of natural phonetic ambiguity in a heterogenous multilingual population of CantoneseEnglish speakers. We test whether these early bilinguals, like second language learners, show an own-voice benefit in word recognition. Moreover, we probe whether the own-voice benefit indeed hinges upon recognition of ones own voice. Following prior work (Holmes et al., 2018; Mitterer et al., 2020), some cues to talker identity are manipulated by shifting f0 and formant frequencies (using Praat; Boersma and Weenink, 2020) to limit listeners ability to recognize their own voices. This methodology draws on the observation that manipulating these cues greatly affects the success of self-voice recognition (Xu et al., 2013).

## Materials and methods

The experiment consisted of three parts: a questionnaire about multilingualism, a production task, and a perception task, all of which were completed remotely on participants own electronic devices. All written and verbal instructions were presented in English to accommodate limited Cantonese literacy within the bilingual population at our university.

### Participants

To be eligible for this study, participants were required to self-identify as female, be exposed to both Cantonese and English at or before the age of six, and minimally have the ability to carry out a basic conversation in Cantonese. Only female subjects were invited to participate to minimize between-speaker variation and to allow a more consistent vocal disguise technique (see description of audio manipulation below). Thirty-six female Cantonese-English bilinguals participated in the experiment. While all participants completed the multilingual questionnaire and the production task, the recordings of three participants obtained during the production task were excluded from the perception task due to poor recording quality and interference from background noise. In addition, two participants who completed the production task and questionnaire did not complete the perception task, resulting in 31 subjects who completed all three parts of the study. Appendix Table A1 provides selected summary language information for the 33 participants who completed the production task and for whom a perception experiment was designed. Appendix Table A2 contains additional demographic information about the participant population. Participants reported their languages in order of current self-assessed dominance, along with the age of acquisition of each language, and speaking, listening, and reading proficiencies on a scale from 0 (none) to 10 (perfect). The population is highly multilingual, as is typical of both Cantonese speakers in Cantonese-speaking homelands (e.g., Hong Kong, Guangzhou) and those in the Cantonese-speaking diaspora, which is the convenience sample used in the current study. For example, 27 participants report Mandarin as an additional language, and 16 report French, in addition to small numbers of individuals self-reporting knowledge of other languages. Participants self-reported ages of acquisition indicate that Cantonese was the earliest acquired language (Median = 0, *SD* = 1.3), compared to English (Median = 3, *SD* = 1.9), and Mandarin (Median = 6, *SD* = 4) and French (Median = 9, *SD* = 2.7), the other two most attested languages amongst participants. Participants self-reported significantly higher speaking and listening proficiencies for English (speaking: *M* = 9.3, *SD* = 0.98; listening: *M* = 9.48, *SD* = 0.83) compared to Cantonese [speaking: *M* = 7.15, *SD* = 2.36; listening: *M* = 7.82, *SD* = 1.96; paired *t*-test for speaking: *t*(32) = 4.38, *p* = 0.0001; paired *t*-test for listening: *t*(32) = 4.11, *p* = 0.0003]. Mandarin was the language with the next highest self-reported proficiency across participants, though it was not a language reported by all participants, and self-reported speaking [unpaired *t*-test: *t*(54) = 2.65, *p* = 0.01] and listening [unpaired *t*-test: *t*(54) = 2.7, *p* = 0.009] skills were higher for Cantonese than Mandarin (speaking: *M* = 5.5, *SD* = 2.5; listening: *M* = 6.4, *SD* = 2.1). Participants current place of residence was in English-dominant communities in Canada and the United States, as shown in Appendix Table A2.

Participants were compensated with gift cards equivalent to 5 CAD for the production task, 5 CAD for the questionnaire, and 10 CAD for the perception task. Participants were recruited through the UBC community and social media.

### Materials

#### Multilingual language questionnaire

Participants completed an online survey that presented questions from the Language Experience and Proficiency Questionnaire (LEAP-Q; Marian et al., 2007) and the Bilingual Language Profile (BLP; Gertken et al., 2014). Both resources were designed to gain a better understanding of language profiles of bilingual and multilingual speakers by including questions relating to individuals language history, usage, attitudes and self-rated proficiency. Additionally, general questions pertaining to participants biographical information were included in this questionnaire. This survey was administered in English.

#### Production stimuli

Stimuli for the production task included monosyllabic Cantonese words, presented as pictures accompanied by English translations. All pictures were hand-drawn by the researcher and presented in black and white so that no single picture was especially salient to subjects (see Appendix Figure A2 for the complete set of visual stimuli). The word list was composed of 22 minimal pairs targeting seven segmental contrasts (see Appendix Table A3 for the complete production word list). Three of the lexical items served as minimal pair to more than one other item, hence the number of unique words totaled 41 (and not 44) for the 22 minimal pairs. The lexical items involved word initial consonants 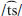, 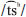, and /s/ and vowel contrasts 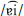 and 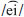, // and 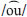, 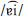 and 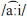, 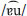 and 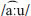, and // and /a:/. Target sounds were selected based on their presence in Cantonese and absence in English such that the selected contrasts would show variability across proficiency ranges in the Cantonese-English bilingual community. For example, three of the vowel contrasts chosen are distinguished by vowel length, a feature that is not lexically contrastive in English. The stimuli were designed to consist of all high level tone (T1) words to control for differences in tone that may cause unwanted variability in production or confusion in perception task performance. The words were chosen to be familiar to Cantonese speakers with potentially limited vocabularies due to largely using Cantonese as a home language in an English-dominant region and had meanings that could be easily represented in pictures. Pictures, as opposed to Chinese characters, were used both in the production and perception tasks to accommodate participants who have limited literacy skills.

#### Perception stimuli

A subset of the stimuli words used in the production task were featured in the perception task. These consisted of 13 minimal pairs featuring five vowel contrasts: 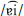 and 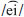, // and 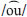, 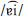 and 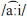, 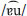 and 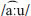, and // and /a:/, which are presented in their character and Jyutping transliterations and English glosses in Table 1. The same pictures corresponding to these target words from the production experiment were used in the perception task. The manipulation of the audio stimuli for the perception experiment is described below.

**TABLE 1:** *Perception Stimuli* arranged by minimal pair.

Chinese Character	English Gloss	Jyutping Romanization	Chinese Character	English Gloss	Jyutping Romanization
	chicken	gai1		machine	gei1
	chicken	gai1		street	gaai1
	to wave	fai1		to fly	fei1
	many	do1		knife	dou1
	song	go1		tall	gou1
	comb	so1		beard, moustache	sou1
	ball	bo1		pot	bou1
	to squat	mau1		cat	maau1
	autumn	cau1		to copy	caau1
	cough	kat1		card	kaat1
	heart	sam1		shirt	saam1
	west	sai1		to waste	saai1
	turtle	gwai1		well-behaved	gwaai1

Note that *chicken* is used in two minimal pairs.

### Production task

#### Procedure

For the production task, participants first watched a video tutorial (made by the first author) on how to record themselves producing the list of target words. This video included a familiarization phase for participants to learn the intended referents of the picture stimuli. For each target word, participants would hear a Cantonese word and see its corresponding picture and English translation. Afterward, participants were instructed to download Praat (Boersma and Weenink, 2020) and record themselves using the built-in microphone of their personal electronic devices at a sampling frequency of 44,100 Hz. Participants accessed a .pdf file containing the picture stimuli and were asked to verbally label the target words in Cantonese, given the picture and English translation as they proceeded through the randomized list at their own pace. Each picture was shown twice to elicit two productions of each word, for a total of 82 productions. Lastly, participants were asked to verbally describe a picture of a busy park scene in Cantonese, in as much detail as they wanted. Participants saved their recordings according to their anonymous participant ID number and uploaded their recordings to Dropbox.

#### Segmentation

Words of the minimal pairs were segmented from recordings using Praat (Boersma and Weenink, 2020). Recordings from three participants were excluded from this process due to poor recording quality. From the productions of the remaining 33 speakers, nine speakers had at least one word excluded for a total of 15 words excluded from analyses due to incorrect labeling of the picture stimuli. The removal of one item entailed the removal of two, as the minimal pair was removed from that individuals set.

Because stimuli words were produced in isolation, word-initial stops /b/, /d/, /g/, /k/ and /k^w^/ were identified as beginning with the stop burst, starting as an abrupt change in amplitude in the waveform and ending with the onset of quasi-periodic activity of the following vowel. The offset of the labialized voiceless velar stop /k^w^/ was identified as a change in the waveform from a simpler periodic pattern to a more complex periodic pattern of a vowel. In this set of stimuli, the only word-final stop was /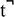/. The end boundary of this unreleased stop was identified as the same point as the end of its preceding vowel. Fricatives /s/ and /f/ were identified in waveforms as aperiodic or random patterns indicating frication noise. Affricates 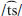 and 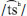 were identified as beginning with a stop burst and ending with the offset of frication noise, signaling the end of the fricative. Aspirated alveolar affricates showed a period of high amplitude frication followed by a period of lower amplitude frication and the boundaries for aspiration were annotated using low amplitude frication as a cue. One participant produced target words intended to contain word-initial aspirated alveolar affricates with voiceless fricatives instead. For these productions, the onset and offset of the aspirated alveolar affricate 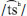 were marked at the same points as the beginning and end of aspiration shown in the waveform. The onset of nasals /m/, /n/ and // were identified at the point of a most discrete change in amplitude in the waveform. The offset of the nasal consonants in word-initial position were indicated by a sudden increase in intensity at the beginning of the following vowel. Another cue used to identify this boundary was the change from a simple waveform pattern with lower frequencies, characteristic of nasal consonants, to a more complex pattern with both high and low frequencies, characteristic of vowels. Likewise, the opposite change in intensity and opposite shift in waveform patterns indicated boundary of the word-final nasal //. All word and sound boundaries were placed as closely as possible to zero crossings to prevent auditory distortions resulting from discontinuities at the beginnings and ends of sound intervals. Words in all 22 minimal pairs were segmented, although only the subset of words comprising 13 minimal pairs were used in the perception task. Target words were saved into their own files, while target sounds were trimmed into files with 25 ms buffers at the onset and offset of sounds in preparation for acoustic analysis.

#### Grouping voices

Acoustic analyses served to group minimal pairs into five groups (Groups A, B, C, D, and E) reflecting how discretely speakers produced the contrast between the two words of each minimal pair. We will refer to this measure as contrastiveness, as it denotes the acoustic difference between target sounds in minimal pairs, but does not necessarily imply speaker proficiency or production accuracy. Because of the considerable amount of individual variation observed between minimal pairs within vowel contrasts, a given talkers group assignment was done separately for each minimal pair. This means that a speaker was not, for example, categorized as a Group A speaker, but her productions for a particular minimal pair may have been assigned to Group A, while her productions for other minimal pairs may be in another contrastiveness Group.

To determine contrastiveness we first estimated formant trajectories with samples every two seconds for each vowel using Fast Track (Barreda, 2021), a formant tracker plug-in via Praat (Boersma and Weenink, 2020). The frequency range was set at 5,0007,000 Hz to reflect a speaker of medium height (Barreda, 2021), as all participants in our study were female adults.

Formant trajectories were then converted from Hertz to the Bark scale to better reflect auditory processing (Traunmller, 1990). With the obtained Bark-scaled formant trajectories, we then performed a discrete cosine transform (DCT) which yielded three primary coefficients for F1 and F2. The three coefficients corresponded to the mean of the formant, the slope of the formant and the curvature of the slope. In addition to these six dimensions, we also measured vowel duration as a seventh dimension in which speakers could potentially show distinctiveness in production. While not all seven dimensions may be used to contrast the target vowels in our minimal pairs, we did not exclude any particular parameter to avoid making any *a priori* claims about the relative importance of these cues for contrastiveness for this bilingual population. We centered, scaled and calculated Euclidean distances for each talkers minimal pair along all seven dimensions.

**FIGURE 1:**
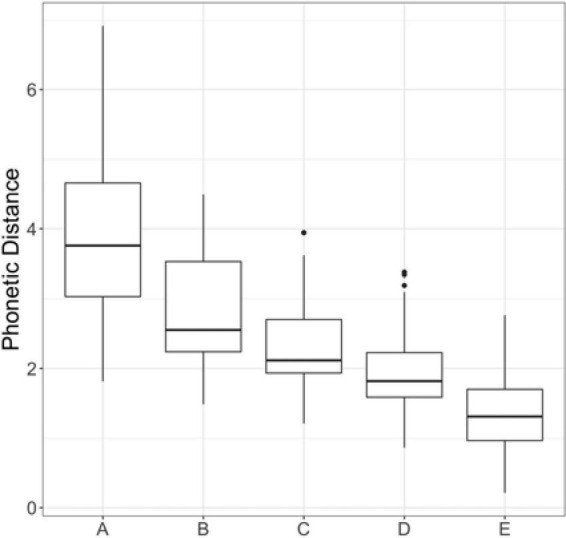
Box-and-whisker plot of phonetic distance between minimal pairs for utterances in the five contrastiveness groups.

Lastly, for each minimal pair, we organized speakers according to the contrastiveness of their productions. This was done by ranking the Euclidean distances for each minimal pair and using the rankings of each to form minimal pair-specific group assignments, in which a greater Euclidean distance indicated a more distinctive production. Within each minimal pair, we formed five groups, ranging from A (most contrastive) to E (least contrastive), consisting of five to seven different voices; thus, for each minimal pair, each group had 5-7 different voices. The groups were manually adjusted to be approximately equally sized, as some talkers were missing tokens and therefore would not be presented with that particular minimal pair in their individualized perception experiment. Figure 1 is a box-and-whisker plot presenting the phonetic distance or contrastiveness range for the productions in each of the five contrastiveness groups.

Each subject was presented with a perception experiment, described below, featuring their own productions and the productions of other members of their contrastiveness group, for each minimal pair. Therefore, the number of different unfamiliar voices heard by each participant varied according to their group memberships.

### Perception task

#### Audio manipulation

For the perception experiment, recordings segmented into isolated words were altered to change female voices into male-like voices using the Change-Gender function in Praat (Boersma and Weenink, 2020). This application lowered the fundamental frequency (f0) and formant frequencies of the original productions by multiplying these dimensions by factors specific to each speaker. Modulation of these parameters have been shown to influence the accuracy of self-voice recognition (Xu et al., 2013) and previous studies have successfully disguised voices using the Change-Gender function (Holmes et al., 2018; Mitterer et al., 2020). For speakers in the current study, the multiplication factors for f0 and formant frequencies ranged from 0.55 to 0.75 (mean = 0.62) and 0.79 to 0.83 (mean = 0.81) respectively. Pitch range parameters were adjusted as necessary to ensure accurate pitch tracking. Following Mitterer et al. (2020), the manipulations started with scaling the f0 by 0.59 and the formants by 0.82, which were the average manipulations made by Mitterer et al. (2020). From there, the actual values for each talker were adjusted by ear to achieve a good-sounding disguise. The specific by-talker adjustments are reported in Appendix Table A4. Finally, the target stimuli were RMS-amplitude normalized to 65 dB and mixed in continuous speech-shaped noise, created from the spectral profiles of the participants speech samples, at a signal-to-noise ratio (SNR) of +5 dB to increase the difficulty of the task. This particular SNR was determined through piloting to achieve high accuracy, but prevent ceiling performance.

#### Procedure

The same speakers who completed the production task were invited to complete the perception task several months later, which was administered online using jsPsych (de Leeuw, 2015). This perception experiment was a two-alternative forced-choice lexical identification task featuring the acoustically altered recordings described above. For each trial, participants heard an isolated Cantonese word produced either by themselves or another speaker along with two pictures on the left and right sides of the screen, representing the appropriate Cantonese minimal pair. Participants were required to choose the picture corresponding to the word they heard by pressing the keys F or J for the left and right sides of the screen, respectively. Participants responses advanced the program to the next trial. Three practice trials were provided. Audio stimuli were presented at a comfortable listening level and participants completed a headphone check prior to beginning the experiment (Woods et al., 2017). There were four repetitions of each token for a total of 560688 trials for each participants personalized experiment [up to 26 items (e.g., 13 minimal pairs) a range of 57 speakers in each by minimal pair group 4 repetitions of each token]. Trials were fully randomized across four blocks between which participants were offered a self-paced break. At the end of the experiment, participants were asked if they recognized their own voice throughout the experiment, to which they selected yes or no on the screen. The perception experiment was completed on participants own electronic devices and took approximately 3540 min to complete. Participants were asked to complete the task in a quiet place.

## Results

To remove extremely fast and extremely slow responses, button presses logged under 200 ms and over 5000 ms were removed from the data, eliminating just under 2% of responses. Participants responses on the perception task were scored as either correct or incorrect depending on whether listeners chose the picture corresponding to the intended word. These accuracy data were analyzed using a Bayesian multilevel regression model in Stan (Gabry and Cenovar, 2021) using brms (Brkner, 2018) in R (R Core Team, 2021). The accuracy of each response (correct word identification or not) was analyzed as the dependent variable with Voice Match (other voice, own voice), Trial number (centered and scaled), and Contrastiveness Group (Groups AE) as independent variables. Voice Match and Group, Trial and Group, and Trial and Voice Match were included as interactions. There were random slopes for Voice Match and Trial by participant. Given that most items were other voice items, Voice Match was treatment coded (with Other Voice as the reference level) and Contrastiveness Group was forward-difference coded using the coding matrices package (Venables, 2021), which compares each level in Contrastiveness Group to the adjacent level. The model family was Bernoulli and we specified weakly informative normally distributed priors that were centered at 0 for the intercept and population-level parameters. The intercept and population-level parameters had standard deviations of 5 and 2.5, respectively, following recommendations for accuracy data in Coretta (2021). Correlations used the LKJ prior with a value of 2. The models were fit with 4000 iterations (1000 warm-up) with four chains for the Hamiltonian Monte-Carlo sampling. All R-hat values were below 1.01 and Bulk ESS values were all high, suggesting the model was well mixed. The median posterior point estimates and the 95% credible interval (CrI) is reported for all parameters and interactions. An effect is considered compelling if 95% of the posterior distribution for a parameter does not include 0. An effect is considered to have weak evidence if the credible interval includes 0, but the probability of direction is at least 95%. These interpretation practices follow recommendations in Nicenboim and Vasishth (2016).

**FIGURE 2:**
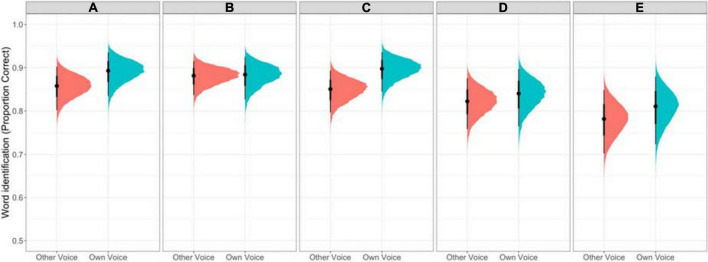
Proportion of correct responses in the perception task for the five acoustic contrastiveness groups presented as fitted draws from the posterior fit of the model. Panels **AE** represent the five contrastiveness groups from most contrastive **(A)** to least contrastive **(E)**. Responses to both own voice and other voices are included.

The model results are reported in Table 2. The intercept indicates that listeners were very good at the task, reliably identifying the intended lexical item [ = 1.66, 95% CrI = [1.32, 2.02], Pr( 0) = 1]. The model results provide compelling evidence for a benefit in processing ones own (disguised) voice [ = 0.23, 95% CrI = [0.06, 0.42, Pr( 0) = 99.5%]. This result is visualized in Figure 2, which presents the fitted draws from the posterior fit of the model for the own-voice effect by Contrastiveness Group.

**TABLE 2:** Summary of the posterior distribution modeling word recognition accuracy with posterior means and the 95% Credible Interval, along with the probability of direction for each effect.

Parameter		95% CrI	Probability of direction
Intercept	1.66	[1.32, 2.02]	100%
Voice Match (Own Voice)	0.23	[0.06, 0.42]	99.5%
Trial	0.07	[0.01, 0.14]	98.22%
Group A vs. B	0.21	[0.36, 0.06]	99.72%
Group B vs. C	0.27	[0.13, 0.41]	100%
Group C vs. D	0.21	[0.07, 0.34]	99.84%
Group D vs. E	0.26	[0.13, 0.39]	100%
Voice Match Group A vs. B	0.31	[0.09, 0.70]	93.69%
Voice Match Group B vs. C	0.41	[0.77, 0.04]	98.60%
Voice Match Group C vs. D	0.31	[0.04, 0.68]	95.55%
Voice Match Group D vs. E	0.04	[0.37, 0.28]	60.03%
Trial Group A vs. B	0.08	[0.06, 0.22]	86.60%
Trial Group B vs. C	0.04	[0.17, 0.09]	73.05%
Trial Group C vs. D	0.03	[0.15, 0.09]	68.46%
Trial Group D vs. E	0.04	[0.15, 0.08]	73.08%
Voice Match Trial	0.03	[0.10, 0.17]	65.33%

An effect of trial suggests that listeners accuracy improved across the course of the experiment [ = 0.07, 95% CrI = [0.01, 0.14], Pr( 0) = 98.22%]; the CrI for all interactions of Trial with the Contrastiveness Group contrasts overlap substantially with 0, suggesting that this cross-experiment improvement was not specific to a particular Group. The Voice Match by Trial interaction also overlapped with 0, indicating there is no evidence that the improvement in word recognition across the course of experiment was better or worse for ones own voice or other voices.

Comparisons of adjacent Contrastiveness Groups generally present compelling evidence that higher proficiency groups perform more accurately on the word identification task [Group B vs. C: = 0.27, 95% CrI = [0.13, 0.41], Pr( 0) = 100%; Group C vs. D: = 0.21, 95% CrI = [0.07, 0.34]; Pr( 0) = 99.84%; Group D vs. E: = 0.26, 95% CrI = [0.13, 0.39], Pr( 0) = 100%] with the exception of Group B outperforming Group A [ = 0.21, 95% CrI = [0.36, 0.06], Pr( 0) = 99.72%]. Two interactions involving Voice Match and Group merit attention. There is compelling evidence for an effect that Group B showed less of an own-group advantage than Group C [ = 0.41, 95% CrI = [0.77, 0.04], Pr( 0) = 98.60 %] and there is weak evidence that Group D showed less of an effect than Group C [ = 0.31, 95% CrI = [0.04, 0.68], Pr( 0) = 95.6%].

## Discussion

This experiment tested an own-voice advantage for word recognition in Cantonese for Cantonese-English early bilinguals. Words were presented in speech-shaped noise at +5 dB SNR to make the task challenging enough to inhibit ceiling performance. Listeners were more accurate at identifying difficult vowel contrasts if they were (vocally disguised) self-produced items compared to items produced by other individuals who manifested the phonological contrast to a similar degree. This was true despite an individuals own voice being disguised, suggesting that the own-voice word recognition benefit leverages linguistic representations that exist in a normalized representational space, as opposed to relying on an exact acoustic-auditory match to ones natural acoustic patterns. Items were organized by the degree of phonetic distance for the phonological contrast into what are labeled contrastiveness groups. There was strong evidence that Group C showed more of an own-voice benefit than Group B and weak evidence that Group C showed a greater own-voice benefit than Group D. Group B was exceptional in stepping out of the anticipated order in overall accuracy. While it was generally the case that groups with higher contrastiveness performed more accurately on the word identification task, Group B out-performed Group A, the highest contrastiveness group. A possibility for why those in Group B were so outstanding may relate to imperfection in our method of calculating acoustic distance, which included acoustic dimensions that are likely not core cues to contrast, though this is speculation. We note that the overall pattern was that the own-voice benefit was robust across contrastiveness groups and word recognition accuracy decreased as contrastiveness was reduced. The contrastiveness groups relate to the degree of distinctiveness of speakers productions, which in turn may relate to speaker proficiency. Like the finding in Eger and Reinisch (2019), however, the own-voice benefit does not seem to hinge on proficiency.

Word recognition accuracy improved over the course of the experiment with participants own voices and other voices. Although subjects heard their own voice more often than any single other voice in the experiment, the proportion of correct responses increased across trials for all voices. Altogether, this suggests that the observed self-advantage was not simply due to listeners hearing their own voice more than other voices throughout the task. The improvement across the experiment was likely due to participants adapting to the noise, which masked the speech to inhibit ceiling performance.

Our ability to determine whether listeners explicitly heard their own voice was based on an explicit self-assessment. A subset of participants reported hearing their own voice in the experiment (*n* = 9), but we cannot (a) confirm that positive responses to this question were not a function of positive response bias or (b) rule out that other listeners did not implicitly hear their own voices. While we follow previous work in our implementation of the voice disguise (Holmes et al., 2018; Mitterer et al., 2020), an individuals voice identity is available in other spectral and temporal patterns. Speakers vary in terms of their unique voice profiles (Lee et al., 2019; Johnson et al., 2020) and listeners exploit different acoustic cues for talker identification (Van Lancker et al., 1985; Lavner et al., 2000). Schuerman et al. (2015, 2019) did not find support for an own-voice advantage within an individuals first language when presenting noise-vocoded speech, a type of degradation in which many spectral cues important to talker identification are severely reduced, though Schuerman (2017) finds some evidence for an own-voice benefit for word recognition in sentences for speech in noise, which better retains talker-specific information. The removal of expected cues to speaker identity does not explain the absence of an own voice-benefit in those studies, however, as voice recognition and speech recognition are separate, but connected systems (for an overview see Creel and Bregman, 2011). Listeners show an intelligibility benefit for familiar voices even when those voices are made unfamiliar, indicating that the familiarity benefit does not rely on explicit recognition of a voice (Holmes et al., 2018).

The prevalent theory in voice representation is that talkers voices are represented according to prototypes. According to the prototype theory, each stimulus is compared to a representative or central member of its category; stimuli that better approximate the prototype will be more easily perceived as belonging to the category (Lavner et al., 2001). Under this interpretation, talker identification relies on the storage and retrieval of identities based on a set of features deviating from the prototype. As previous studies have shown, the acoustic dimensions used to characterize different voices are often talker-specific (Van Lancker et al., 1985; Lavner et al., 2000). Voices that deviate more from the prototype are perceived as more distinct and thus, the more distant a speakers acoustic features are from the central model, the easier the speaker is to be identified (Lavner et al., 2001; Latinus et al., 2013). This may partially explain the variance in participants self-reports of hearing their own voices in the current study despite our attempt to disguise vocal identity. Those who successfully identified themselves may have had voices that deviated more from the average template and were therefore easier to recognize. Researchers have proposed that the prototype is an average, commonly encountered, yet attractive voice (Lavner et al., 2001; Latinus et al., 2013; Lavan et al., 2019). Accordingly, this voice should be representative of the listeners language input and environment, and people of the same linguistic community would be expected to share a similar template (Lavner et al., 2001). The implications for having a voice that approximates listeners community prototypes with regards to a benefit in word recognition needs to be explored further. In Schuerman et al. (2015, 2019) studies, researchers identified a statistically average speaker among the subjects in their studies to represent the average of the linguistic community. When presented with noise-vocoded speech, native Dutch listeners in their studies showed better recognition of words produced by the statistically average speaker in their sample than the listeners themselves. This implies that the benefit of a prototypical voice may extend beyond the benefit of hearing ones own voice for word recognition.

The core finding in the current work is that listeners were more accurate in recognizing minimal pairs produced in their own (disguised) voice than recognizing the realizations of other speakers who maintain similar degrees of phonetic contrast for the same minimal pairs. These findings with Cantonese-English bilinguals, a population which was targeted to leverage the heterogeneity in pronunciation variation within a native speaker population, replicating and extending the findings for second language learners (Eger and Reinisch, 2019). We present evidence of an own-voice benefit for work recognition, like Eger and Reinisch, but this benefit is seen when voices were disguised and the majority of individuals did not report consciously recognizing their own masked voice.

Crucially, the own-voice advantage in word recognition suggests that the phonetic distributions that undergird phonological contrasts are heavily shaped by ones own phonetic realizations, extending the importance of self-produced items beyond real-time self-monitoring (e.g., Howell et al., 2006; Niziolek and Guenther, 2013). Online compensation for altered auditory feedback indicates that auditory self-monitoring leads to immediate, though incomplete, adjustments in speech production. Importantly, the magnitude of these adjustments is yoked to whether the auditory feedback suggests a linguistic contrast is threatened (Niziolek and Guenther, 2013). This suggests a coupled relationship between perception and production where an individuals representational space for perception and recognition align with the distributional pool available for that individual in production. Many frameworks posit some degree of connection between perception and production with theoretical models differing in terms of how parsimonious perception and production repertoires are, amongst other theoretical differences related to the actual representational space (e.g., Liberman and Mattingly, 1985; Fowler, 1996; Johnson, 1997; Goldinger and Azuma, 2004). Certainly, listeners abilities to perceive phonetic detail is connected to their abilities to produce contrasts (e.g., Werker and Tees, 1984), but does not wholly limit it (e.g., Schouten et al., 2003). Listeners are well attuned to the distribution of phonetic variation within their speech communities, particularly when that phonetic variation has social value (e.g., Johnson et al., 1999; Hay et al., 2006; Munson et al., 2006; Szakay et al., 2016). A fully isomorphic production and perception system fails to account for how listeners adapt to novel input from other speakers without concomitantly changing their own productions (Kraljic et al., 2008). If perception and production exclusively relied on perfectly mapped mental representations, the reorganization of phonetic space or changes in the weighting of acoustic cues due to perceptual learning should also be observed in that individuals productions, but this is not well supported in the existing literature (Schertz and Clare, 2020).

What mechanism accounts for the own-voice benefit? One possibility is that the mere constant auditory exposure to ones own voice, despite the fact that an individual need not attend to their own speech for the purpose of comprehension, bestows such a high level of familiarity that it is privileged in recognition space. Alternatively, it is plausible that the way in which an individual produces a contrast is intimately tied to the way in which the contrast is realized by their most frequent interlocutors such that this manifestation of the contrast realized by the most familiar voices and ones own receives a recognition benefit. This explanation seems unlikely, however, given that second language learners (Eger and Reinisch, 2019) and our early bilingual population show the same own-voice benefit. A third possibility is that while, as described above, perception and production cannot be isomorphic, the yoking of an individuals speech production repertoire and that repertoires mapping in the perceptual space is what benefits an individuals own-voice productions in recognition. This is also an interpretation offered for own-voice recognition by Xu et al. (2013), who suggest that own-voice auditory and motor representations are connected. The representation of perception and action in shared space is at the heart of the common coding hypothesis (Prinz, 1997). Assuming a shared representational space for perception and production, the common coding theory predicts that listeners compare incoming speech signals to their own productions. Therefore, in perceiving ones own voice, recognition is facilitated because the auditory signal aligns with the listeners own productions to a greater degree. Support for this in the recognition space comes from speech-reading. Individuals are better at keyword recognition in sentences when speech-reading silent videos of themselves compared to others (Tye-Murray et al., 2013), in addition to receiving more of an audio-visual boost in noisy conditions with their own videos (Tye-Murray et al., 2015). If a shared representational space accounts for the own-voice benefit, it apparently must be part of a developmental trajectory, however, as Cooper et al. (2018) find no evidence for an own-voice benefit (or an own-mother voice) benefit for word recognition in 2.5 year olds (see also Hazan and Markham, 2004). Toddlers are better at recognizing any adult production (their own mother or a different mother) than recognizing self-produced words or words from another toddler. Infants, however, already use sensorimotor information in speech perception. English-acquiring six-month olds abilities to perceive retroflex and dental stop contrasts is inhibited when a soother blocks tongue movement [Bruderer et al., 2015; see also Choi et al. (2019) for more evidence about the connection between sensorimotor and perceptual processing in infants]. These sets of results suggest that phonemic perception and word-level recognition have different developmental trajectories with respect to the integration of motor and auditory/acoustic information streams. Ultimately, the current study cannot adjudicate between these explanatory mechanisms, but rather provides additional evidence of an own-voice benefit in adult word recognition (Tye-Murray et al., 2013, 2015; Eger and Reinisch, 2019). Multiple threads in the literature do seem to suggest that the integration of production and perceptual representations offers promise in terms of explanatory force.

The proposed mechanism that supports an own-voice benefit in word recognition the integration of motor and acoustic-auditory representations in the linguistic representations used for word recognition is not intended to be unique to L2 speech processing (e.g., Eger and Reinisch, 2019) or the processing of ones less dominant language (e.g., the current work). It may simply be easier to observe the evidence of an own-voice benefit in individuals non-dominant language(s) because it may be more error prone. Individuals native or dominant languages also, of course, exhibit within- and cross-talker variability (e.g., Newman et al., 2001; Vaughn et al., 2019). It is important to note that while there is strong statistical evidence in support of an own-voice benefit in the current work, the effect is small. An own-voice benefit is also not mutually exclusive with a benefit for a typical voice that represents the prototype or central tendency of the local speech community (e.g., Schuerman, 2017). While listeners are highly adaptable, leveraging any available information in the signal to recognize words, it is important that work in this area use spectrally rich speech samples, as some adverse listening conditions, like noise-vocoded speech, do not encode the full array of spectral information listeners typically have access to in spoken language processing. A degraded signal may encourage listeners to engage in different processing strategies.

While the own-voice benefit for word recognition was statistically robust, some participants did appear to perform less accurately on their own voices. If some aspects of word recognition are related to community averages or prototypes, these individual differences could be accounted for by considering how distant a particular individual is from the prototype. For example, participants exemplifying a self-benefit may better approximate the prototype, while those performing worse with their own voices may deviate more from the prototype relative to other speakers in their group. This reasoning aligns with the Schuerman et al. (2015, 2019) explanation for the benefit bestowed by the statistically average voice. A shared representation for an average speaker in a heterogenous bilingual population presents a challenge, however. In multilingual speech communities where individuals vary in proficiency and language use patterns, which voices are used to form prototypes for which languages? That is, are there separate prototypes, for example, for apparent native speakers of Cantonese and apparent native speakers of English, with separate prototypes established for individuals whose voices suggest a variety of Cantonese-accented English or English-accented Cantonese? What is the representational space for a speaker who experiences speaking and listening to all of these codes in different contexts? We note the nebulous nature of this space, not to discount its importance, but rather to encourage further research that can tackle the complexities in phonetic variation that are experienced by multilingual individuals.

Our recruitment criteria specified exposure to Cantonese from an early age, at or prior to age six. This lumps very early and early acquisition and both simultaneous and sequential bilinguals all in a single group. This may ultimately not be a uniform population. Exposure to a language from birth has implications for pronunciation patterns. For example, Amengual (2019) examined the lenition rates of phrase-initial voice stops and approximants in the Spanish of simultaneous Spanish-English bilinguals, early sequential Spanish-then-English bilinguals, and late Spanish learners (with English as a first language). The simultaneous bilinguals and late learners patterned together. Given that exposure to English from birth unifies these two groups, these results suggest that early exposure to English has the potential to shape pronunciation patterns in adulthood, similar to previous suggestions for perception (e.g., Sebastin-Galls et al., 2005). The developmental trajectory out of the sensitive period, however, is gradual, and what exactly is the appropriate age delimiter for a particular linguistic representation, pattern, or process is yet to be determined (see, for example, Flege, 1999; Werker and Tees, 2005; Cargnelutti et al., 2019).

## Conclusion

Early CantoneseEnglish bilinguals exhibited an own-voice benefit for word recognition in Cantonese even when self-recognition of their own voice was masked by a vocal disguise. These results complement the evidence indicating an own-voice benefit in second language speakers (Eger and Reinisch, 2019). The own-voice benefit despite overt recognition of ones own voice suggests a coupled relationship between the motor representations and the multidimensional acoustic-auditory representations that support word recognition.

## Data availability statement

The listener data supporting the conclusions can be made available by the authors upon request, as that is what aligns with the approved Ethics protocol.

## Ethics statement

The studies involving human participants were reviewed and approved by University of British Columbias Behavioural Research Ethics Committee. The patients/participants provided their written informed consent to participate in this study.

## Author contributions

SC and MB contributed to the conception and design of the study. SC prepared the stimuli and wrote the first draft. MB performed the statistical analyses and wrote sections of the manuscript. Both authors approved the submitted version.
