# Supplementary material for: The own-voice benefit for word recognition in early bilinguals
Source: Front Psychol. 2022 Sep 2;13:901326. doi: 10.3389/fpsyg.2022.901326 (PMC9478475; doi:10.3389/fpsyg.2022.901326)
Supplement: Supplementary file 1 [file Data_Sheet_1.pdf]

Appendix A

Table A1. Participant language information. Participants' languages in order of dominance, along with age of acquisition and self-rated speaking, listening, and reading proficiencies for each language. Note the age of acquisition question was likely interpreted by some participants as the age of onset of speaking, given that some participants do not report age 0 for any language. Proficiencies range from 0-none to 10-perfect.

Participant	Order of Language Dominance	Age of Acquisition	Speaking Proficiency	Listening Proficiency	Reading Proficiency
1	English	0	10	10	10
	Cantonese	0	5	7	5
	Mandarin	5	5	6	5
	French	10	1	0	1
2	English	3	10	10	10
	Cantonese	0	10	10	10
	Mandarin	9	7	8	7
	French	10	3	3	3
3	English	2	10	10	10
	Cantonese	0	4	5	4
	French	9	1	1	1
	American Sign Language	22	2	2	2
4	English	4	10	10	10
	Cantonese	0	7	8	7
	Mandarin	16	2	4	2
5	English	3	9	9	9
	Cantonese	1	8	8	8
	Mandarin	5	4	5	4
	French	10	2	2	2
6	English	1	9	10	9
	Mandarin	1	7	8	7
	Cantonese	6	2	3	2
	Hakka	1	2	3	2
7	English	0	10	10	10
	French	5	7	7	7
	Cantonese	0	3	4	3
	Japanese	15	2	1	2
8	Cantonese	0	10	10	10
	Mandarin	6	8	9	8
	English	3	7	9	7
	Shanghainese	17	1	3	1
	Korean	15	1	2	1
9	English	2	10	10	10
	Cantonese	0	10	10	10
	Mandarin	3	9	9	9
	French	9	7	7	7
10	English	4	10	10	10
	Cantonese	0	8	8	8
	Mandarin	0	5	5	8

	French	9	3	3	3
11	English	1	10	10	10
	French	3	8	8	8
	Cantonese	0	8	8	8
	Mandarin	4	3	6	3
12	Cantonese	0	10	10	10
	English	3	7	8	7
	Mandarin	13	1	3	1
	German	18	0	0	0
	French	7	0	0	0
13	English	3	9	9	9
	Cantonese	0	9	9	9
	Mandarin	6	7	7	7
14	English	4	10	10	10
	Cantonese	3	10	9	10
15	English	0	10	10	10
	Cantonese	0	3	3	3
16	English	1	10	10	10
	Cantonese	1	7	8	7
	Mandarin	7	6	7	6
17	English	4	10	10	10
	Cantonese	0	7	7	7
	French	7	1	1	1
18	English	4	10	10	10
	Cantonese	0	7	9	7
	Mandarin	14	6	8	6
	Korean	16	6	7	6
	Canadian French	6	4	4	4
	Japanese	12	2	2	2
19	Mandarin	1	9	8	9
	Cantonese	0	8	8	8
	English	3	7	7	7
20	English	2	9	9	9
	Cantonese	2	7	8	7
	Mandarin	6	2	5	2
21	English	3	10	10	10
	Cantonese	1	7	9	7
	Mandarin	6	6	7	6
	Spanish	8	2	3	2
	French	10	1	1	1
22	Cantonese	0	9	10	9
	English	2	8	9	8
	Mandarin	6	7	8	7
	Korean	18	3	3	3
	Japanese	10	2	2	2
	Spanish	12	2	2	2
23	English	4	10	10	10
	Cantonese	0	7	8	7
	French	8	5	6	5
	Mandarin	7	2	3	2
24	English	6	10	10	10
	Cantonese	4	5	5	5
	Village dialect	1	5	5	5

	Mandarin	8	4	4	4
	French	14	2	2	2
	Arabic	19	1	1	1
25	Cantonese	0	10	10	10
	English	5	10	10	10
	Mandarin	6	9	9	9
	French	12	4	2	4
26	Mandarin	0	9	9	9
	English	6	9	9	9
	Cantonese	0	3	6	3
	Spanish	14	3	2	3
27	English	1	10	10	10
	Cantonese	1	6	9	6
	French	9	3	3	3
	Japanese	19	1	1	1
28	English	0	10	10	10
	French	6	8	8	8
	Cantonese	0	8	8	8
	Mandarin	8	5	5	5
	Spanish	15	2	3	2
29	Cantonese	0	9	9	9
	English	6	8	7	8
	Mandarin	6	4	5	4
	Japanese	13	2	3	2
30	English	0	9	9	9
	Cantonese	0	7	7	7
	German	28	7	7	7
	Mandarin	13	6	5	6
	Spanish	16	2	2	2
31	English	2	8	9	8
	Cantonese	0	9	9	9
	Mandarin	6	7	8	7
	Japanese	15	5	5	5
32	English	7	9	10	9
	Cantonese	0	4	7	4
	Japanese	16	2	3	2
	Mandarin	7	1	2	1
33	Cantonese	0	9	9	9
	English	3	9	9	9
	Mandarin	3	7	9	7
	Japanese	14	6	7	6

Table A2. Selection of additional participant demographic information. Participants' age at the time of study, their level of education, and current place of residence are provided.

**Participant**	**Age at Time of Study**	**Level of Education**	**Place of Residence**
1	25	Graduate degree	Vancouver, BC, Canada

2	23	Undergraduate degree	BC, Canada
3	24	Undergraduate degree	Vancouver, BC, Canada
4	23	Undergraduate degree	Coquitlam, BC, Canada
5	23	Undergraduate degree	Vancouver, BC, Canada
6	24	Graduate degree	Hamilton, ON, Canada
7	22	Undergraduate degree	Vancouver, BC, Canada
8	25	Undergraduate degree	Richmond, BC, Canada
9	22	Undergraduate degree	Vancouver, BC, Canada
10	23	Undergraduate degree	Richmond, BC, Canada
11	25	Graduate degree	Westmount, Quebec, Canada
12	36	Graduate degree	Richmond, BC, Canada
13	29	Undergraduate degree	Vancouver, BC, Canada
14	20	Undergraduate degree	Vancouver, BC, Canada
15	30	Graduate degree	Vancouver, BC, Canada
16	19	Undergraduate degree	Richmond, BC, Canada
17	27	Graduate degree	Vancouver, BC, Canada
18	19	Undergraduate degree	Richmond, BC, Canada
19	23	Undergraduate degree	Vancouver, BC, Canada
20	26	Graduate degree	Vancouver, BC, Canada
21	24	Undergraduate degree	Victoria, BC, Canada
22	23	Graduate degree	Vancouver, BC, Canada
23	26	Undergraduate degree	Toronto, ON, Canada
24	31	Undergraduate degree	Honolulu, HI, USA

25	22	Undergraduate degree	Vancouver, BC, Canada
26	21	Undergraduate degree	BC, Canada
27	23	Graduate degree	Vancouver, BC, Canada
28	23	Undergraduate degree	Vancouver, BC, Canada
29	23	Diploma	Vancouver, BC, Canada
30	40	Graduate degree	Honolulu, HI, USA
31	22	Undergraduate degree	Vancouver, BC, Canada
32	39	Undergraduate degree	Burnaby, BC, Canada
33	23	Undergraduate degree	Richmond, BC, Canada

Materials

Table A3.
Production Word List

Chinese Character	English Gloss	Jyutping Romanization
雞	chicken	gai1
機	machine	gei1
街	street	gaai1
揮	to wave	fai1
飛	to fly	fei1
多	many	do1
刀	knife	dou1
歌	song	go1
高	tall	gou1
梳	comb	so1
鬚	beard/mustache	sou1
波	ball	bo1
煲	pot	bou1
踎	to squat	mau1
貓	cat	maau1
秋	autumn	cau1
抄	to copy	caau1
咳	cough	kat1
咭	card	kaat1
心	heart	sam1
衫	shirt	saam1
西	west	sai1
嘥	to waste	saai1
龜	turtle	gwai1
乖	well-behaved	gwaai1
揸	drive	zaa1
叉	fork	caa1
遮	umbrella	ze1
車	car	ce1
鐘	clock	zung1
蔥	onion	cung1
追	to chase	zeoi1
吹	to blow	ceoi1

尖	sharp	zim1
簽	to sign	cim1
獅	lion	si1
黐	to stick	ci1
星	star	sing1
蜻	dragonfly	cing1
沙	sand	saa1
鬆	loose	sung1

Figure A1 Picture Stimuli.
Picture stimuli for the production task with characters, Jyutping, and an English gloss. A subset of these items was used in the perception task.

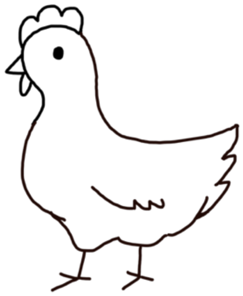

雞 gai1 “chicken”

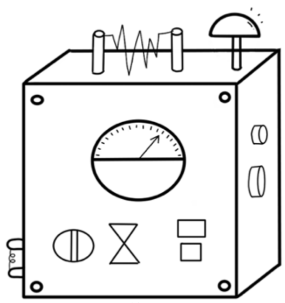

機 gei1 “machine”

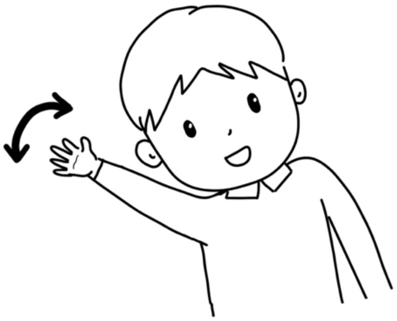

揮 fail "to wave (one's hand)"

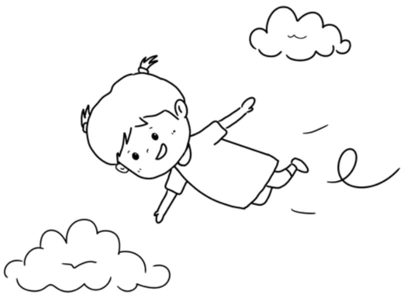

飛 feil "to fly"

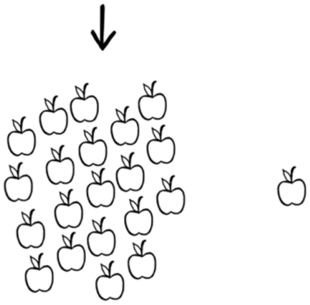

多 dol "many"

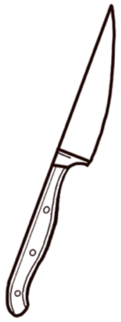

刀 doul "knife"

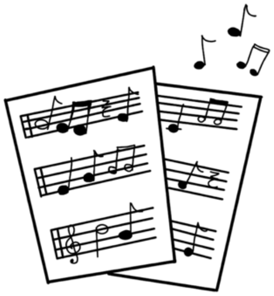

歌 gol "song"

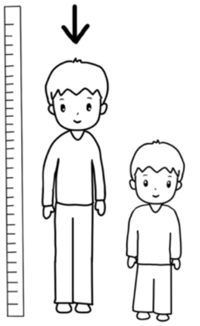

高 goul "tall"

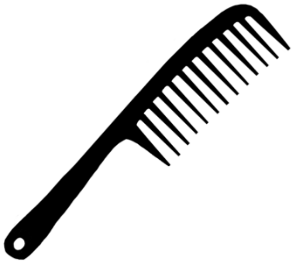

梳 sol "comb"

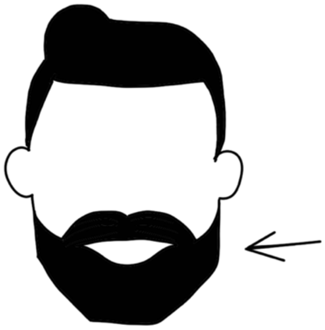

鬚 soul "beard/mustache"

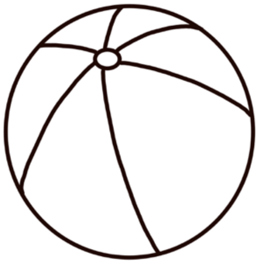

波 bol “ball”

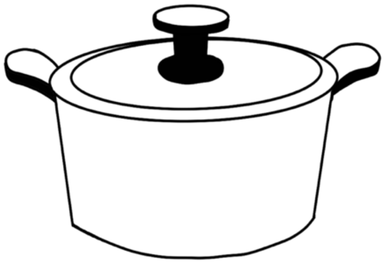

煲 bou1 “pot”

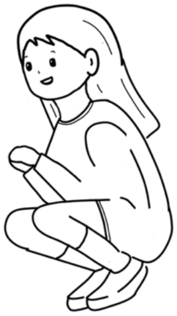

踎 mau1 “to squat”

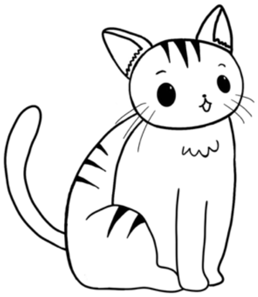

貓 maau1 “cat”

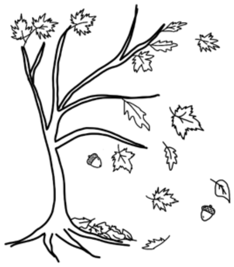

秋 caul “autumn”

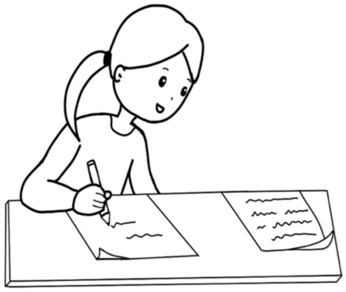

抄 caaul “to copy”

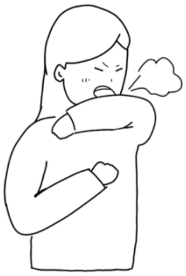

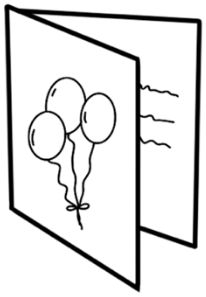

咯 kat1 “to cough”	咭 kaat1 “card”
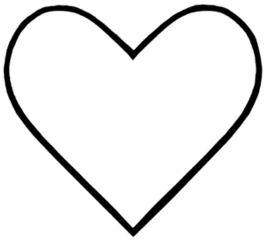	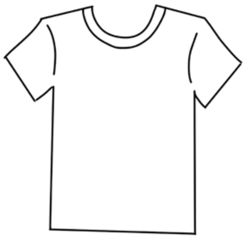
心 sam1 “heart”	衫 saam1 “shirt”
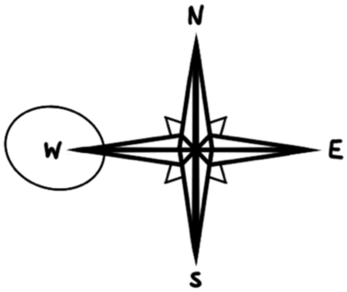	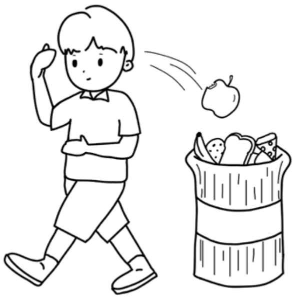
西 sai1 “west”	嘥 saai1 “to waste”
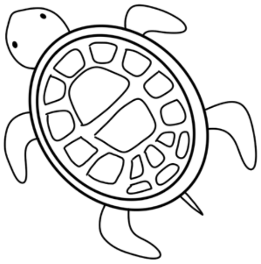	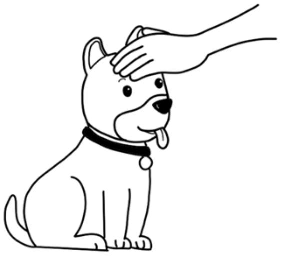
龜 gwai1 “turtle”	乖 gwaai1 “well-behaved”
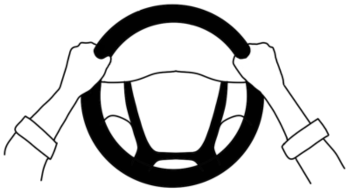	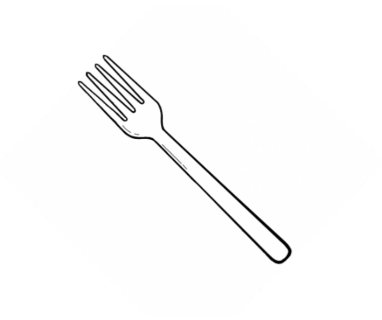

揸 zaa1 “to drive”

叉 caal “fork”

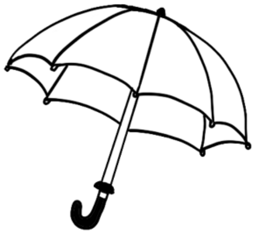

遮 zel “umbrella”

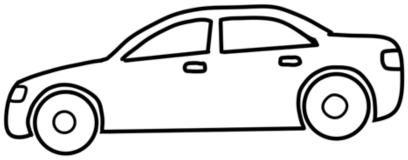

車 cel “car”

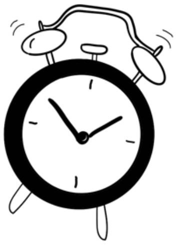

鐘 zungl “clock”

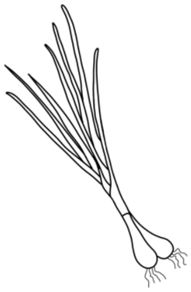

葱 cungl “green onion”

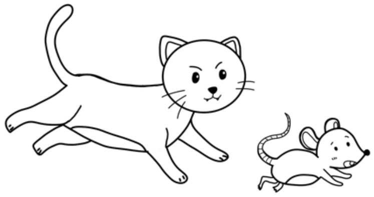

追 zeoil “to chase”

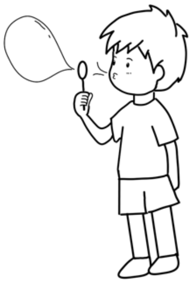

吹 ceoil “to blow”

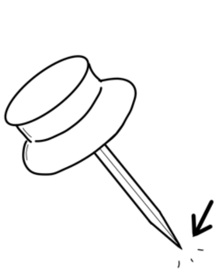

尖 ziml “sharp”

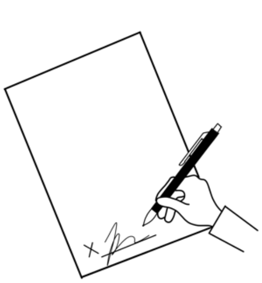

簽 ciml “to sign”

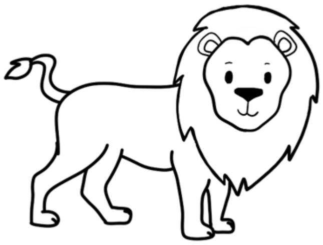

獅 sil “lion”

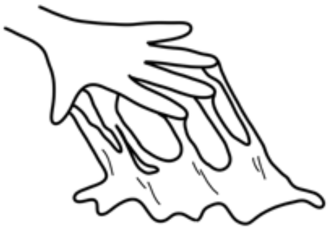

黐 ci “to stick”

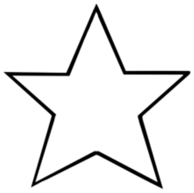

星 singl “star”

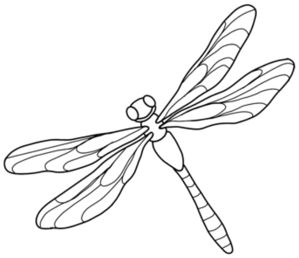

蜻 cingl “dragonfly”

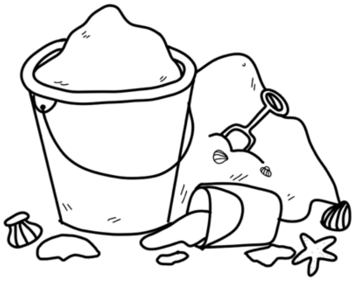

沙 saal “sand”

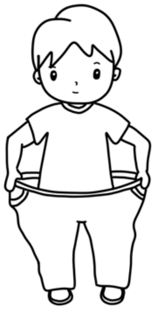

鬆 sungl “loose”

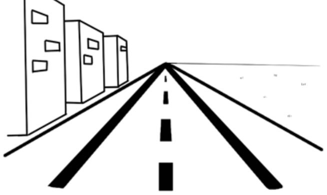

街 gaail “street”

Figure A2 The following pictures were used for practice trials in the perception task.

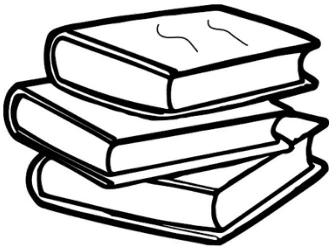

書 syu1 “book”

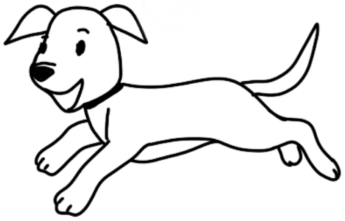

狗 gau2 “dog”

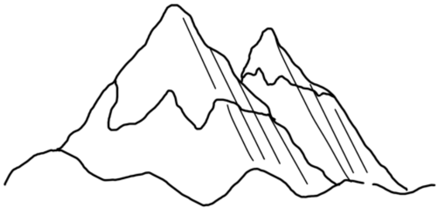

山 saan1 “mountain”

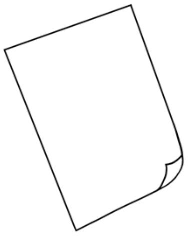

紙 zi2 “paper”

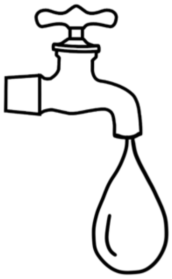

水 seoi2 “water”

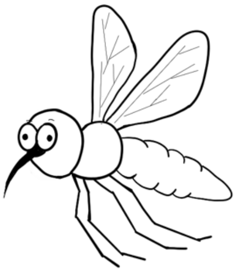

蚊 man1 “mosquito”

Perception Task Audio Manipulation

Table A4

Modification parameters and pitch measurement parameters used with the Change-Gender function in Praat.

Participant	Pitch range factor	Formant shift ratio	Pitch floor	Pitch ceiling
1	0.57	0.8	150 Hz	300 Hz
2	0.75	0.83	150 Hz	300 Hz
3	0.65	0.79	150 Hz	300 Hz
4	0.6	0.83	150 Hz	300 Hz
5	0.65	0.8	150 Hz	500 Hz
6	0.6	0.82	150 Hz	300 Hz
7	0.65	0.8	150 Hz	500 Hz
8	0.75	0.83	150 Hz	300 Hz
9	0.6	0.82	150 Hz	300 Hz
10	0.6	0.8	150 Hz	300 Hz
11	0.55	0.8	150 Hz	300 Hz
12	0.65	0.82	150 Hz	300 Hz
13	0.55	0.8	150 Hz	300 Hz
14	0.6	0.8	150 Hz	300 Hz
15	0.6	0.83	150 Hz	500 Hz
16	0.55	0.79	150 Hz	300 Hz
17	0.6	0.82	150 Hz	300 Hz
18	0.55	0.79	150 Hz	300 Hz
19	0.6	0.83	150 Hz	300 Hz
20	0.65	0.8	150 Hz	300 Hz
21	0.57	0.81	150 Hz	300 Hz
22	0.58	0.8	150 Hz	300 Hz
23	0.7	0.83	150 Hz	300 Hz
24	0.69	0.83	150 Hz	300 Hz

25	0.57	0.79	150 Hz	300 Hz
26	0.56	0.79	150 Hz	300 Hz
27	0.58	0.79	150 Hz	300 Hz
28	0.6	0.82	150 Hz	300 Hz
29	0.65	0.83	150 Hz	300 Hz
30	0.68	0.8	150 Hz	300 Hz
31	0.6	0.8	150 Hz	300 Hz
32	0.63	0.81	150 Hz	300 Hz
33	0.7	0.81	150 Hz	300 Hz
